# All eyes on gas exchange: impact of controlled arterial hypertension on cardiopulmonary function and the effects of high-intensity interval training

**DOI:** 10.1097/HJH.0000000000004394

**Published:** 2026-07-16

**Authors:** Fabian Schwendinger, Jonathan Wagner, Christoph Hauser, Eva Fleischlin, Justin Carrard, Arno Schmidt-Trucksäss, Henner Hanssen, Lukas Streese

**Affiliations:** a Division of Sports and Exercise Medicine,; b Division of Exercise and Environmental Physiology, Department of Sport, Exercise and Health, University of Basel, Basel; c Sport and Youth Health Unit, Children and Adolescent Surgery, Women's, Maternal and Child Health Department; d Sport and Exercise Medicine Centre, Musculoskeletal Medicine Department, Lausanne University Hospital; e Institute of Sport Science, University of Lausanne, Lausanne, Switzerland; f Faculty of Health Care, Niederrhein University of Applied Sciences, Krefeld; g Department of Nephrology, Medical Faculty, Heinrich-Heine-University Düsseldorf, Germany

**Keywords:** aerobic exercise, exercise capacity, exercise intervention, hypertension, physical activity

## Abstract

**Introduction:**

Arterial hypertension (AH) impairs vascular function which, in turn, may reduce cardiopulmonary function. However, the impact of AH on cardiorespiratory fitness (CRF) and its potential mechanisms remains insufficiently investigated. We compared cardiopulmonary function during exercise testing (CPET) between adults with and without AH (part 1) and evaluated the effects of high-intensity interval training (HIIT) in adults with AH on submaximal cardiopulmonary CPET parameters (part 2).

**Methods:**

Thirty-eight adults with AH and 19 controls without AH partook in the study. Adults with AH were randomized into either HIIT or a control group. CPET assessed CRF and submaximal exercise parameters, including the oxygen uptake efficiency slope (OUES), V̇E/V̇CO_2_ slope, PETCO_2_, V̇O_2_/work rate (WR) slope, and O_2_ pulse. The HIIT intervention consisted of triweekly sessions over 8 weeks.

**Results:**

Adults with AH had a 6.5 ml kg^−1^ min^−1^ (95% confidence interval: 1.7–11.3) lower CRF, a steeper V̇E/V̇CO_2_ slope (moderate-large effect size), lower PETCO_2_ (large effect size), flatter V̇O_2_/WR slope (moderate effect size), and lower O_2_ pulse (small-moderate effect size) than adults without AH. HIIT improved CRF and altered certain cardio-circulatory parameters in AH, including OUES (small-large effect size), V̇O_2_/WR slope (small-moderate effect size, low precision) and O_2_ pulse (small-moderate effect size). However, effects of HIIT on pulmonary-vascular parameters were inconclusive, with large variability observed in the V̇E/V̇CO_2_ slope and PETCO_2_.

**Conclusion:**

Adults with controlled AH might have lower CRF, indicating limitations in pulmonary-vascular and cardio-circulatory organ systems. A short-term 8-week HIIT enhances CRF and cardio-circulatory function, however, evidence is limited for improvements in pulmonary-vascular function.

## Graphical abstract

**Figure:**
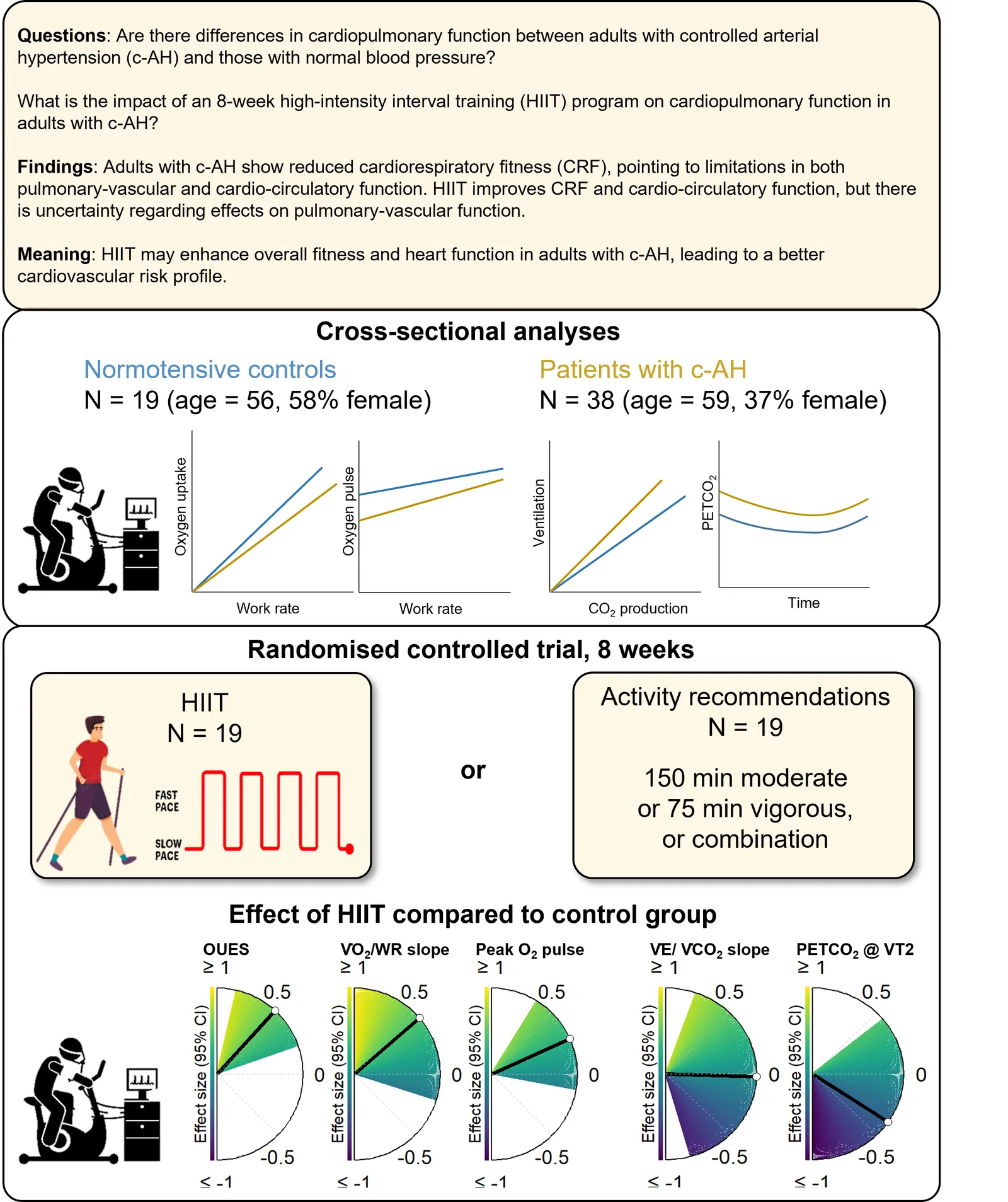

## INTRODUCTION

Since 2000, the prevalence of arterial hypertension (AH) is projected to increase by 60% globally, reaching a total of 1.56 billion cases by 2025 [1,2]. Cardiorespiratory fitness (CRF) is a vital sign and an important risk predictor for various non-communicable diseases [3,4]. In individuals with AH, exercise training has been shown to increase CRF [5]. Moreover, higher CRF in adults with hypertension is protective against carotid atherosclerosis[6] and reduces mortality risk [7].

Cardiopulmonary exercise testing (CPET) is the gold standard for determining CRF by measuring maximum oxygen uptake (V̇O_2max_) [3,4]. Beyond assessing CRF, CPET offers a comprehensive evaluation of the mechanical-ventilatory, pulmonary-vascular, cardio-circulatory, and peripheral organ systems [8]. This enables researchers and clinicians to identify and differentiate between various mechanisms that may limit CRF, providing valuable insights for targeted interventions and treatment strategies [8]. However, little is known about how CPET parameters differ between adults with normal BP and those with hypertension. Dekleva *et al.*[9] reported lower CRF, reduced O_2_ pulse, and slightly worse ventilatory efficiency in adults with grade I and II AH. Extending these analyses to additional CPET parameters reflecting cardio-circulatory and pulmonary-vascular function would be valuable for understanding the full impact of AH on the cardio-pulmonary system.

A large body of evidence indicates that aerobic high-intensity interval training (HIIT) effectively increases CRF in healthy individuals and diseased populations, including those with pre- and established AH across the age spectrum [5,10]. However, little is known about how HIIT impacts submaximal CPET parameters.

Thus, we aimed to investigate:

1. differences in CPET parameters reflecting cardio-circulatory and pulmonary-vascular organ function between adults with controlled AH (c-AH) and controls with normal blood pressure (BP).
2. the effect of an 8-week HIIT intervention on CPET parameters reflecting cardio-circulatory and pulmonary-vascular organ function in adults with c-AH.

## MATERIALS AND METHODS

### Study designs (cross-sectional and randomized controlled trial)

The HyperVasc study consisted of two parts. Part 1 was designed to compare 40 adults with c-AH to 20 controls with normal BP cross-sectionally.

In part 2, all 40 adults with c-AH were randomized to an intervention group and a control group. Randomization was done post-baseline assessments by an independent, blinded researcher. The study was conducted at the Department of Sport, Exercise and Health in Basel, Switzerland, following the Helsinki Declaration. All participants provided written informed consent. The study was approved by the Ethics Committee of Northwestern and Central Switzerland (EKNZ-2021-00086) and registered on ClinicalTrials.gov (NCT04763005). Data collection ran from March 2021 to December 2021. Further details are available in the study protocol [1].

### Eligibility criteria

Individuals aged 40-70, previously diagnosed with controlled grade 1 AH (BP ≤159/99 mmHg) and individuals aged 40–70 without hypertension (BP ≤129/84 mmHg) [1], were recruited via local newspaper advertisements. Exclusion criteria include any cardiovascular medication other than antihypertensives for adults with hypertension, history of cardiovascular (other than AH), pulmonary or chronic inflammatory diseases, active smoking, a BMI ≥30 kg/m^2^, exercise-limiting orthopedic issues, or changes in antihypertensive treatment during the study.

### Blood pressure measurement

Office BP was measured using the Boso Carat Professional (BOSCH + SOHN GmbH u. Co. KG, Jungingen, Germany) and categorized according to the 2018 European Society of Cardiology and European Society of Hypertension guidelines. Twenty-four-hour ambulatory BP was measured using the Mobil-O-Graph device (I.E.M GmbH, Aachen, Germany).

### Cardiopulmonary exercise testing

Gas exchange was measured breath-by-breath using the Cortex MetaMax 3B (Cortex Biophysik GmbH, Germany) during a ramp test on a cycle ergometer at >60 rpm under controlled conditions. The ramp protocol was chosen based on the participant's estimated CRF, beginning with a 3-min warm-up at 10, 20, or 50 W [11]. Subsequently, the workload was increased linearly by 10, 15, or 20W min^−1^. After test cessation, participants cycled for three additional minutes at the same intensity as during the warm-up [11]. Heart rate (HR) was measured by 12-lead electrocardiography (Custo med GmbH, Ottobrunn, Germany). Peak oxygen uptake (V̇O_2peak_) was determined as the highest 30 s mean during the exercise phase. In the absence of a V̇O_2_ plateau, defined as an increase in V̇O_2_ from the second last to the last minute during exercise of <125 ml min^−1^[12], maximum exhaustion was verified by achieving a maximum respiratory exchange ratio (RER_max_) of 1.1 for ages 40–59 and 1.06 for ages 60–69 [13]. Three individuals (one at baseline and two at post-examination) did not exhibit a V̇O_2_-plateau nor fulfil the RER_max_ criterion. These were handled in sensitivity analyses described below.

1. We calculated the following slopes from the beginning of the ramp phase up to ventilatory threshold 1 (VT1), ventilatory threshold 2 (VT2), and exercise cessation:
2. Oxygen uptake efficiency slope (OUES) as the slope of a linear regression fitted to predict oxygen uptake (V̇O_2_) from log-transformed ventilation (log[V̇E]).
3. Ventilatory efficiency for carbon dioxide slope (V̇E/V̇CO_2_) as the slope of a linear regression fitted to predict ventilation from CO_2_ production.
4. Oxygen uptake – work rate slope (V̇O_2_/WR) as the slope of a linear regression fitted to predict V̇O_2_ from WR.
5. End-tidal partial pressure of CO_2_ (PETCO_2_) was reported at VT1 and VT2. O_2_ pulse (O_2_ uptake/heart rate in ml/beat) was calculated at VT1, VT2, and V̇O_2peak_.

### Exercise intervention and physical activity recommendations

Participants in the intervention group engaged in eight weeks of triweekly, supervised HIIT sessions, consisting of 4 × 4-min intervals of fast uphill walking at 80–95% of maximum heart rate (HR_max_), interspersed with 3-minute periods of active recovery at 60–70% of HR_max_. Exercise intensity was controlled based on heart rate using the Polar H7 chest strap and M400 watch (Polar, Kempele, Finland). The control group followed physical activity recommendations based on European Society of Cardiology guidelines and were advised to document their PA in a diary. After four weeks, we assessed their well-being via phone calls. More details are available in the study protocol [1].

## STATISTICAL ANALYSIS

Analyses were done in R version 4.2.0.

*Cross-sectional analyses*: Linear regression models estimated the difference between adults with hypertension and healthy controls in CPET parameters of interest. Models were adjusted for age (continuous), sex (dichotomous), and skeletal muscle mass (continuous). For models with non-normal residuals, bootstrapping with 10 000 replicates produced estimates and bias-corrected, accelerated 95% confidence intervals (CIs), robustly addressing distribution irregularities. We conducted sensitivity analyses to explore medication as potential confounder by including renin-angiotensin system inhibitors and calcium channel blockers (binary) as additional covariates in the main models (see Figure 2, Supplemental Digital Content).

*RCT analyses*: Analyses of the intervention data were conducted based on the intention-to-treat principle. Linear mixed models (R-package ‘lme4’ version 1.1-33) were used to estimate the effect of group allocation on changes over time in the outcome variables. Age (continuous) and sex (dichotomous) were included as covariates as specified *a priori*[1]. Models included random intercepts to address variability in performance trajectories over time among the participants. Estimates of the product term between group and time with 95% CIs are reported. In addition to reporting results on the original scales, variables in the models were scaled to facilitate comparisons across different physiological metrics and effect sizes were interpreted as small (≥0.1–<0.3), medium (≥0.3–<0.5) and large (≥0.5) on the *Z*-scale [14]. The interpretation of our findings is based on the magnitude of an effect in combination with its uncertainty/precision (95% CI).

Partial least squares regression analysis was conducted to determine whether changes in OUES were primarily driven by the cardio-circulatory system or the pulmonary-vascular system, identifying key physiological variables most strongly associated with OUES changes.

## SAMPLE SIZE CALCULATION

The main outcome of this study was the differences of microvascular health between adults with AH and healthy controls as well as the exercise intervention effects on microvascular health. To reach a power of 95% with a two-sided significance level of 0.05, a total sample size of 46 participants was needed [1].

## RESULTS

**FIGURE 1:**
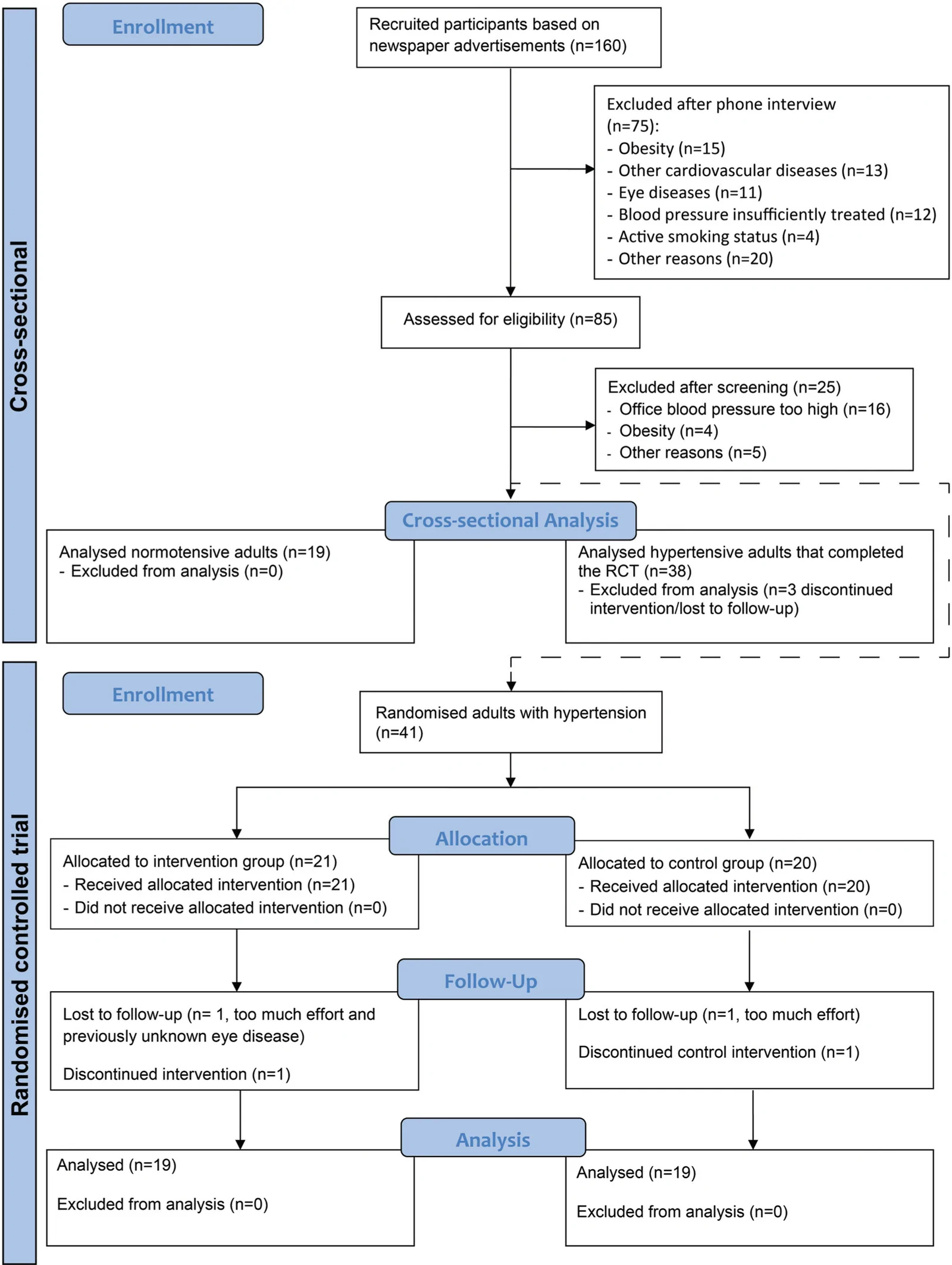
Flow of study participants for the cross-sectional comparison of controls with normal blood pressure to adults with arterial hypertension as well as the randomized controlled trial with adults with arterial hypertension.

The flow of study participants is shown in Fig. 1. We included 19 normotensive controls and 38 adults with c-AH in the cross-sectional analyses. There 38 adults with c-AH were 1:1 randomized to a control and intervention group for the randomized controlled trial. Cohort characteristics by group are shown in Table 1 and Table 1, Supplemental Digital Content. Further characteristics of 24-h BP measurements are available in Tables 1 and 2, Supplemental Digital Content.

**TABLE 1:** Cohort characteristics for normotensive controls and adults with hypertension stratified intervention/control group

	Normotensive controls (*n* = 19)	Control group (*n* = 19)		Intervention group (*n* = 19)	
Variable		Pre	Post	Pre	Post
Age, years	56 (53, 59)	62 (58, 65)	–	56 (54, 61)	–
Sex, % female	58	42	–	32	–
Height, m	1.71 (1.65, 1.80)	1.76 (1.66, 1.80)	–	1.76 (1.62, 1.82)	–
Weight, kg	67.1 (62.2, 76.4)	78.2 (66.7, 85.7)	75.5 (65.8, 85.3)	82.3 (65.0, 88.5)	82.8 (64.0,86.6)
Body fat, %	14.9 (12.3, 20.1)	20.5 (17.6, 25.0)	20.3 (15.6, 24.8)	20.5 (24.6, 31.6)	17.8 (15.8, 22.6)
Smoking status, *n*					
Non-smokers	17	18	–	19	–
Ex-smokers	2	1	–	0	–
Medication, yes					
Beta-blockers	0	3	–	0	–
RAAS inhibitors	0	11	–	11	–
Calcium channel blockers	0	6	–	13	–
24-h BP, sys/dia, mmHg	119 (114, 123)/81 (72, 83)	127 (121, 134)/84 (77, 88)	126 (118, 133)/78 (75, 89)	132 (123, 133)/85 (81, 89)	126 (123, 132)/85 (80, 87)
Time since arterial hypertension diagnosis (years)	–	6 (3.5, 11.0)	–	9 (3.5, 12.5)	–
RER_max_	1.11 (1.08, 1.16)	1.09 (1.05, 1.1)	1.06 (1.01, 1.10)	1.09 (1.06, 1.13)	1.07 (1.05, 1.09)
APMHR, HR_max_ % of 210 − age	110 (105, 116)	112 (109, 116)	110 (107, 113)	112 (103, 115)	109 (102, 111)
*P*_max_, W	224 (172, 313)	214 (176, 226)	214 (169, 231)	208 (163, 266)	225 (175, 267)
Absolute V̇O_2peak_, l min^−1^	2.53 (1.97, 3.44)	2.60 (2.08, 2.90)	2.63 (2.04, 2.85)	2.50 (2.08, 3.15)	2.92 (2.14, 3.12)
Relative V̇O_2peak_, ml kg^−1^ min^−1^	36.0 (32.0, 46.0)	33.0 (30.0, 38.0)	31.0 (29.5, 38.0)	33.0 (27.5, 36.0)	35.0 (33.25, 39.5)

Data are presented as median (25th, 75th percentile). See Table 1, Supplemental Digital Content for a descriptive comparison of adults with vs. without AH without stratification by intervention group. The ex-smokers category includes participants that have not smoked since 3, 8, or 21 years.

APMHR, age-predicted maximum heart rate; BP, blood pressure; peak oxygen uptake; *P*_max_, maximum power, RAAS, renin–angiotensin–aldosterone system inhibitors; RER_max_, maximum respiratory exchange ratio; sys, systolic, V̇O_2peak_.

### Cross-sectional: adults with hypertension vs. healthy controls

**FIGURE 2:**
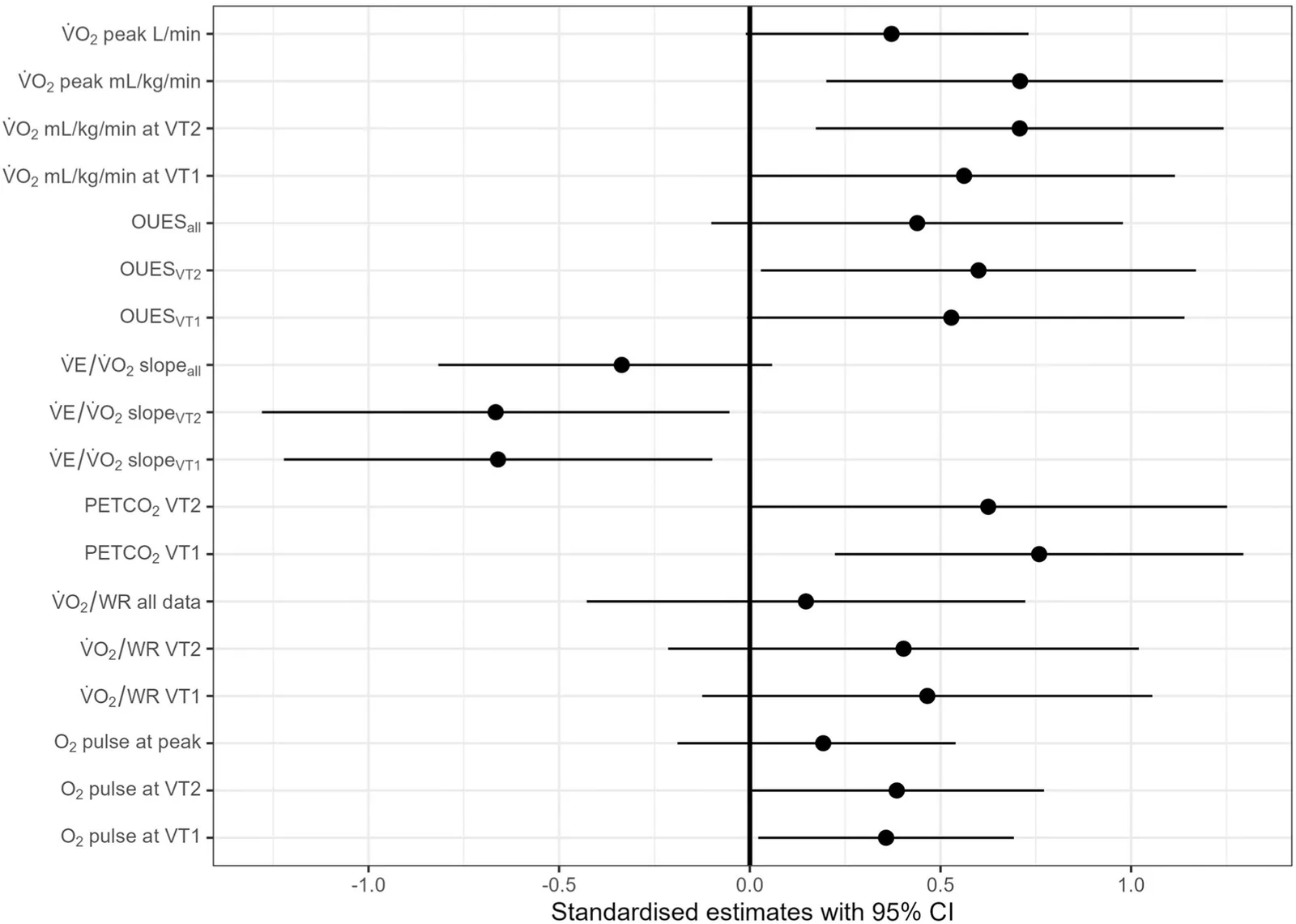
Estimates are on the *Z*-scale and may be interpreted as small (≥0.1–<0.3), medium (≥0.3–<0.5) and large (≥0.5) [14]. Positive *Z*-scores indicate higher values in normotensive adults than in adults with hypertension and vice versa. O_2_ pulse, oxygen pulse; OUES, oxygen uptake efficiency slope; PETCO_2_, partial pressure of end-tidal carbon dioxide; V̇E/V̇CO_2_, ventilatory equivalent for carbon dioxide; V̇O_2_/WR, oxygen uptake to WR ratio; V̇O_2peak_, peak oxygen uptake; VT1, ventilatory threshold 1; VT2, ventilatory threshold 2.

**FIGURE 3:**
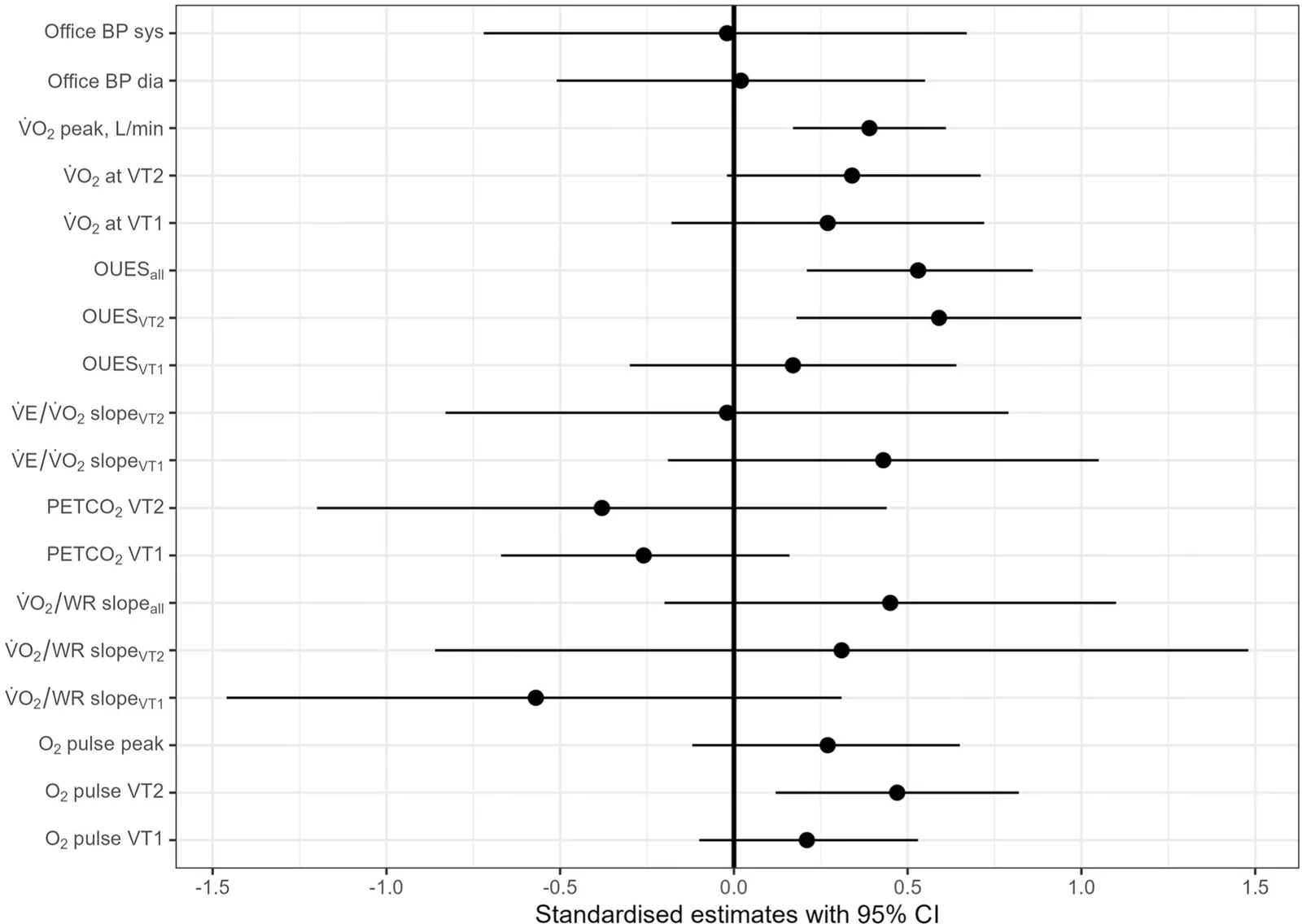
Effect of high-intensity interval training on cardiopulmonary exercise testing parameters. Estimates are on the *Z*-scale and may be interpreted as small (≥0.1–<0.3), medium (≥0.3–<0.5) and large (≥0.5) [14]. Positive *Z*-scores indicate that high-intensity interval training increased the parameter and vice versa in adults with hypertension. BP, blood pressure; dia, diastolic; O_2_ pulse, oxygen pulse; OUES, oxygen uptake efficiency slope; PETCO_2_, partial pressure of end-tidal carbon dioxide; sys, systolic; V̇E/V̇CO_2_, ventilatory equivalent for carbon dioxide; V̇O_2_/WR, oxygen uptake to WR ratio; V̇O_2peak_, peak oxygen uptake; VT1, ventilatory threshold 1; VT2, ventilatory threshold 2.

Between-group differences in gas exchange parameters and slopes are shown in Figs. 2 and 3 and Table 3, Supplemental Digital Content.

### Randomized controlled trial: HIIT-induced changes in adults with controlled arterial hypertension

Antihypertensive medication intakes were comparable between adults with c-AH randomized either to the control or the intervention group. Adults allocated to the intervention group took mainly renin-angiotensin-aldosterone system inhibitors (*n* = 15, 79%) or calcium channel blockers (*n* = 4, 21%). Adults allocated to the control group took mainly renin–angiotensin–aldosterone system inhibitors (*n* = 13, 68%), calcium channel blockers (*n* = 3, 16%), or beta-blockers (*n* = 3, 16%).

### Blood pressure

There was little evidence of the effect of HIIT on systolic and diastolic 24-h ambulatory BP, and there was a large uncertainty (number of observations = 76; Table 2).

**TABLE 2:** Intervention effects of high-intensity interval training in adults with arterial hypertension

Variable	Control group		HIIT group		Linear mixed model: group-by-time effects	
	Pre	Post	Pre	Post	β (95% CI)	Effect size (95% CI)
BP						
24-h ambulatory BP sys/dia (obs = 76)	128 (7)/82 (7)	126 (9)/81 (7)	129 (10)/85 (7)	127 (8)/83 (6)	−0.2 (−6–6)/0.2 (−4–4)	−0.02 (−0.72–0.67)/0.02 (−0.51–0.55)
Gas exchange slopes						
OUES all (obs = 72)	36.6 (11.9)	35.8 (10.6)	33.1 (7.1)	37.0 (6.0)	4.82 (1.86–7.78)	0.53 (0.21–0.86)
OUES VT2 (obs = 50)	41.9 (13.5)	39.5 (12.2)	34.8 (5.7)	37.7 (7.7)	5.4 (1.7–9.2)	0.59 (0.18–1.00)
OUES VT1 (obs = 72)	38.2 (13.6)	40.7 (13.3)	34.2 (7.0)	38.6 (8.7)	1.88 (−0.30–6.99)	0.17 (−0.30–0.64)
V̇E/V̇CO_2_ VT2 (obs = 48)	31.6 (4.6)	32.5 (4.4)	34.2 (4.6)	35.0 (5.5)	−0.10 (−4.08–3.88)	−0.02 (−0.83–0.79)
V̇E/V̇CO_2_ VT1 (obs = 74)	28.6 (6.6)	26.5 (4.9)	28.7 (3.5)	28.8 (4.9)	2.20 (−0.97–5.36)	0.43 (−0.19–1.05)
V̇O_2_/WR all data (obs = 72)	10.2 (1.7)	10.1 (0.9)	9.8 (0.9)	10.2 (1.3)	0.54 (−0.24–1.33	0.45 (−0.20–1.10)
V̇O_2_/WR VT2 (obs = 48)	10.4 (0.9)	10.4 (0.6)	9.8 (0.8)	10.0 (1.2)	0.30 (−0.83–1.43)	0.31 (−0.86–1.48)
V̇O_2_/WR VT1 (obs = 74)	8.6 (1.4)	9.2 (1.1)	8.7 (1.5)	8.5 (1.5)	−0.79 (−2.01–0.43)	−0.57 (−1.46–0.31)
Gas exchange parameters						
V̇O_2peak_, l/min (obs = 74)	2.60 (0.82)	2.53 (0.75)	2.53 (0.63)	2.75 (0.69)	0.28 (0.12–0.44)	0.39 (0.17–0.61)
V̇O_2_ at VT2 (obs = 50)	2.60 (0.92)	2.52 (0.87)	2.30 (0.63)	2.46 (0.61)	0.24 (−0.02–0.50)	0.34 (−0.02–0.71)
V̇O_2_ at VT1 (obs = 74)	1.57 (0.59)	1.51 (0.43)	1.60 (0.40)	1.67 (0.55)	0.13 (−0.09–0.36)	0.27 (−0.18–0.72)
PETCO_2_ VT2 (obs = 50)	35.2 (4.4)	36.1 (4.6)	35.2 (3.6)	34.5 (4.1)	−1.52 (−4.78–1.74)	−0.38 (−1.20–0.44)
PETCO_2_ VT1 (obs = 74)	38.6 (3.9)	39.3 (3.9)	38.6 (2.5)	38.4 (2.8)	−0.85 (−2.23–0.53)	−0.26 (−0.67–0.16)
O_2_ pulse at V̇O_2peak_ (obs = 74)	16.1 (4.4)	15.8 (4.1)	16.1 (5.4)	17.0 (4.2)	1.20 (−0.52–2.93)	0.27 (−0.12–0.65)
O_2_ pulse at VT2 (obs = 50)	16.3 (5.1)	15.9 (4.7)	15.1 (3.9)	16.6 (4.0)	1.95 (0.49–3.41)	0.47 (0.12–0.82)
O_2_ pulse at VT1 (obs = 74)	13.1 (3.9)	13.2 (3.6)	13.1 (3.9)	14.0 (4.0)	0.80 (−0.40–1.99)	0.21 (−0.10–0.53)

Pre and post values are reported as mean (SD). Analyses were conducted using linear mixed models with random intercepts, including age and sex as covariates, to estimate the effect of group allocation on changes in the outcome over time, reporting group-by-time interaction estimates with 95% confidence intervals.

BP, blood pressure; O_2_ pulse, oxygen pulse; obs, observations; OUES, oxygen uptake efficiency slope; PETCO_2_, partial pressure of end-tidal carbon dioxide; V̇E/V̇CO_2_, ventilatory equivalent for carbon dioxide; V̇O_2_/WR, oxygen uptake to work rate ratio; V̇O_2peak_, peak oxygen uptake; VT1, ventilatory threshold 1; VT2, ventilatory threshold 2.

### Gas exchange slopes

HIIT largely improved OUES up to VT2 and across all exercise data, with 95% CIs indicating small to large effect sizes. Small effects were observed for OUES up to VT1, with 95% CI ranging from negative moderate to large positive effects (Table 2). Moreover, HIIT had negative trivial and positive moderate effects on V̇E/V̇CO_2_ slope up to VT2 and VT1, respectively. The data are also compatible with large negative to large positive effect sizes.

For V̇O_2_/WR slope, HIIT had positive moderate and trivial effects across all data and up to VT2, respectively, with 95% CIs ranging from large negative to large positive effects. However, a negative large effect was seen on V̇O_2_/WR slope up to VT1, with possible CI variations from moderate positive to even larger negative effects.

### Gas exchange parameters

V̇O_2peak_ (in l/min) improved in favor of the HIIT group with a moderate effect size. Our data are also compatible with small to large effect sizes. Likewise, HIIT moderately increased V̇O_2_ at VT2 (in l/min), with effect sizes from trivial to large (Table 2) and V̇O_2_ at VT1 (in l/min) with a small effect size, with confidence intervals indicating possible effects from trivial to large.

HIIT decreased PETCO_2_ at VT2 and VT1, with effect sizes ranging from small to moderate and data compatibility extending from trivial to large effects. Small to moderate increases in O_2_ pulse at maximum, VT2, and VT1 were observed, with 95% CIs ranging from small negative to large positive.

Partial least squares regression analysis revealed that changes in O_2_ pulse (coefficient = 302) and V̇O_2_/WR slope (coefficient = 65) were strongly associated with changes in OUES. In contrast, changes in V̇E/V̇CO_2_ slope were negatively associated (coefficient = −123), while changes in PETCO_2_ at VT2 showed a weaker positive association (coefficient = 10).

### Sensitivity analyses

Excluding three individuals who did not meet the predefined exhaustion criteria yielded comparable results for the intervention's effects on both OUES (0.51, 95% CI [0.16, 0.86]) and V̇O_2_/WR slope (0.45, 95% CI [−0.23, 1.12]) calculated across all exercise data as well as V̇O_2peak_ (0.39, 95% CI [0.17, 0.61]).

## DISCUSSION

Our key findings indicate that adults with c-AH have lower CRF compared to normotensive adults, which is associated with a steeper V̇E/V̇CO_2_ slope, lower PETCO_2_, and flatter V̇O_2_/WR slope and O_2_ pulse during exercise, despite some uncertainty in effect sizes. These results suggest potential limitations in the pulmonary-vascular and cardio-circulatory systems. While HIIT has been shown to improve CRF and specific cardio-circulatory parameters, its impact on pulmonary-vascular parameters remains unclear after 8 week of exercise training. Nonetheless, HIIT may be a promising therapeutic option to improve the cardiovascular risk profile and cardiopulmonary function in adults with c-AH, though the underlying mechanisms warrant further investigation.

### Cross-sectional: adults with controlled arterial hypertension vs. healthy controls

CRF was 6.5 (95% CI: 1.7–11.3) ml kg^−1^ min^−1^ lower in adults with c-AH than normotensive peers. Increasing CRF is an important therapy target to reduce the overall cardiovascular disease risk and manage AH [15,16]. The observed lower CRF in adults with c-AH in this study is associated with an elevated risk of mortality and non-communicable diseases of at least 7.3% and 8.7%, respectively, underscoring its clinical relevance even if the lower end of the 95% CI is considered [4].

By analyzing submaximal CPET parameters, we identified the pulmonary-vascular organ systems as a potential contributor to lower CRF in this population. Specifically, regardless of the calculation method, we consistently found steeper V̇E/V̇CO_2_ slopes in adults with hypertension compared to normotensive controls. This aligns with findings that a steeper V̇E/V̇CO_2_ slope is a predictor of hypertension in patients who underwent surgery for aortic coarctation [17]. The steeper V̇E/V̇CO_2_ slope combined with worse OUES and lower PETCO_2_ values indicates worse ventilatory efficiency and potential ventilation-perfusion mismatching [8,18], a pattern that has previously been documented in patients with hypertension [19]. However, this mismatching might also be due to cardio-circulatory limitations, as both OUES and V̇E/V̇CO_2_ can be affected in case of cardiac dysfunction, e.g. heart failure or coronary artery disease [8,18,20]. Furthermore, we observed signs of cardio-circulatory organ system limitations potentially affecting CRF. A flatter V̇O_2_/WR slope (trivial to moderate effect) and a lower O_2_ pulse (small to moderate effect) may reflect impaired oxygen delivery by the heart and lungs and/or oxygen utilization by the periphery [8,21].

Lifestyle-induced deconditioning likely plays a key role in the abovementioned differences [22]. Additional pathophysiological mechanisms might include left ventricular hypertrophy (prevalent in around 36–41% of individuals with AH) [23], endothelial and microvascular dysfunction [24], and pulmonary hypertension or mitochondrial dysfunction [8,22,25]. Lastly, direct effects of medication on CPET parameters in adults with c-AH should be considered [26]. Yet, to determine the exact mechanisms, further targeted examinations are needed.

The findings above highlight two key aspects. First, differences in submaximal CPET parameters, though generally within the normal range, may suggest an elevated risk for future cardiovascular events if not addressed. Targeted interventions to correct these impairments could be key in managing and reducing cardiovascular disease risk, as well as improving CRF in individuals with c-AH. Second, CRF impairment in c-AH is likely multifaceted, necessitating comprehensive therapeutic strategies addressing cardiovascular and cardiopulmonary function.

### Randomized controlled trial: HIIT-induced changes in adults with controlled arterial hypertension

HIIT has already been shown to improve CRF in adults with AH [5] with our findings supporting this. However, our results go beyond and provide for the first time further insights into the altered organ systems, resulting in improved oxygen delivery/utilization and, ultimately, greater CRF. Of note, we observed distinct changes in the cardio-circulatory organ system, particularly in V̇O_2_/WR slope across all exercise data and O_2_ pulse. The large decrease in V̇O_2_/WR, when calculated up to VT1, could signal improved work economy during low-intensity cycling. Conversely, the moderate increase in V̇O_2_/WR across all exercise data and up to VT2 could indicate better oxygen delivery/utilization during more intense exercise, possibly through greater stroke volume or mitochondrial density/function [8,27]. The small to moderate increase in O_2_ pulse supports this, which again may highlight improved oxygen delivery/utilization [21,27]. This is in line with findings in other patient populations, including cancer [28], coronary heart disease [29], and pulmonary hypertension [30]. Nonetheless, our findings should be interpreted cautiously due to their limited precision. Furthermore, HIIT led to small to large improvements in oxygen uptake efficiency, suggesting enhanced cardiorespiratory function driven primarily by cardio-circulatory changes rather than pulmonary-vascular ones. Partial least squares regression highlighted O_2_ pulse and V̇O_2_/WR slope as the main contributors to these improvements, both being cardio-circulatory parameters (see Supplemental Digital Content). These findings are supported by a recent study showing that HIIT induces adaptations in cardiac function, although the magnitude of these adaptations appears to be attenuated in individuals with AH compared with normotensive populations [31].

However, our findings regarding the effects of HIIT on the pulmonary-vascular organ system are inconclusive. Up to VT1, the V̇E/V̇CO_2_ slope moderately steepened, with effect sizes from small negative to large positive, highlighting variability in ventilatory responses at lower intensities. In contrast, the V̇E/V̇CO_2_ slope exhibited a trivial flattening up to VT2 with low precision, indicating inconsistent impacts on CO_2_ removal. Furthermore, PETCO_2_ exhibited a small to moderate decrease, although effect sizes span from large negative to large positive, suggesting a nuanced and potentially unfavorable effect of HIIT on this parameter. Collectively, our findings suggest that the effects of HIIT on the V̇E/V̇CO_2_ slope and PETCO_2_ are variable and may be influenced by individual differences in response to the training. As such, it is premature to draw firm conclusions from these results. These findings collectively suggest that HIIT may be an effective therapy to improve CRF by counteracting deconditioning and enhancing the cardio-circulatory system. However, the evidence regarding the pulmonary-vascular system remains inconclusive.

### Limitations

This is one of the first studies diving into the extensive CPET data and utilizing it to investigate the differences between normotensive individuals and adults with c-AH as well as the effects of HIIT on various organ system limitations in adults with c-AH. However, it remains challenging to determine the extent to which the observed differences are attributable to underlying pathophysiological mechanisms specific to AH, as opposed to the influence of hypertension medication [32] or deconditioning, particularly given the complex relationship between lifestyle factors such as physical activity and AH. Partial, medication-related residual confounding cannot be excluded. Additionally, the relatively short duration of the 8-week intervention, while sufficient to observe increases in CRF, may be too brief to capture other adaptations fully. Moreover, effect sizes of HIIT on CPET parameters may vary depending on the presence of comorbidities and AH status on the population level. Although not the primary focus of this study, our data showed an increase in systolic office BP, albeit with considerable uncertainty, leaving open the possibility of a slight reduction. In contrast, 24-hour ambulatory BP remained unchanged. These findings differ from those of a meta-analysis of 27 randomized controlled trials, which reported an average reduction of 11 mmHg systolic and 5 mmHg diastolic, with most interventions lasting at least ten weeks. This discrepancy may be attributed to the shorter intervention duration in our study, which explicitly focused on adults with arterial hypertension without other comorbidities. However, this allowed us to specifically examine the effect of exercise on BP. Lastly, a larger cohort could increase the precision of the observed effects and improve the generalizability of the cross-sectional findings.

## CONCLUSION

We demonstrate that adults with c-AH exhibit lower CRF than peers with normal BP, accompanied by indicators of potential pulmonary-vascular and cardio-circulatory organ system limitations, reflected by a steeper V̇E/V̇CO_2_ slope, lower PETCO_2_, and flatter V̇O_2_/WR slope as well as lower O_2_ pulse. While HIIT may effectively improve CRF and enhance specific cardio-circulatory parameters, its impact on pulmonary-vascular function remains inconclusive. These findings suggest that HIIT may serve as both a promising preventive and therapeutic strategy to improve the cardiovascular risk profile in adults with c-AH, though further research is needed to clarify the underlying mechanisms and potential medication effects, particularly in the pulmonary vascular system.

## ACKNOWLEDGEMENTS

Effect size plots in the graphical abstract were generated in R using the EffectVisR package by Schwendinger & Lichtenstein (2024) available on GitHub (https://github.com/FSchwendinger/EffectVisR). Heart and blood vessels icons are from Freepik on Flaticon.

Autors’ contributions: F.S. participated in the concept and design of the article, performed the statistical analyses, interpreted the findings, and wrote the manuscript. J.W. participated in the article's concept, design, review and editing. C.H., E.F., and J.C. supported the study team in data acquisition by performing CPET and medical assessments. A.S.T. supported the study by sharing his clinical and scientific expertise and contributed to data interpretation. H.H. conceptualized the study with L.S., shared his clinical and scientific expertise and contributed to data interpretation. L.S. conceptualized and coordinated the study, and was responsible for micro- and macrovascular assessments and the intervention. All authors revised the manuscript and approved the final version of the manuscript.

Funding: This work was supported by the Forschungsfonds grant from the University of Basel to L.S. The University of Basel was not involved in study design, collection, management, analysis and interpretation of data or writing this manuscript.

Ethics approval statement: The study was approved by the Ethics Committee of Northwestern and Central Switzerland (EKNZ-2021-00086) and registered on ClinicalTrials.gov (NCT04763005).

Patient consent statement: All participants provided written informed consent.

### Conflicts of interest

There are no conflicts of interest.

## Supplementary Material

Supplemental Digital Content
