# Supplementary material for: All eyes on gas exchange: impact of controlled arterial hypertension on cardiopulmonary function and the effects of high-intensity interval training
Source: J Hypertens. 2026 Jul 16;44(10):1881–9. doi: 10.1097/HJH.0000000000004394 (PMC13566482; doi:10.1097/HJH.0000000000004394)
Supplement: Supplementary file 1 [file jhype-44-1881-s001.docx]

**Supplementary digital content**

**Figure 1.** Directed acyclic graph illustrating the assumed causal relationships between hypertension group and cardiopulmonary exercise testing parameters.

**Supplementary Table 1.** Cohort characteristics for normotensive controls and adults with hypertension.

**Supplementary Table 2.** Characteristics of 24-hour blood pressure recording.

**Supplementary Table 3.** Effect for normotensive controls compared to adults with controlled arterial hypertension

**Results of Partial Least Squares Regression.** Contribution of changes in selected submaximal gas exchange parameters to changes in oxygen uptake efficiency slope.

**Figure 2.** Cross-sectional sensitivity analyses.

**Directed acyclic graph**

Figure 1 shows the underlying causal assumptions of the analyses comparing differences in cardiopulmonary exercise testing parameters between normotensive controls and adults with hypertension. Analyses were adjusted for medication intake, age, sex, body mass index, and skeletal muscle mass. We did not adjust for the type of medication as this variable likely constitutes a mediator rather than a confounder because hypertension status may influence the drug class prescribed, which in turn may affect cardiopulmonary exercise testing parameters.

**Figure 1.** Directed acyclic graph illustrating the assumed causal relationships between hypertension group and cardiopulmonary exercise testing parameters. Abbreviations: BMI, body mass index; CPET, cardiopulmonary exercise testing; meds, medication.

**Cohort characteristics**

| **Supplementary Table 1.** Cohort characteristics for normotensive controls and adults with controlled arterial hypertension. | | |
| --- | --- | --- |
| **Variable** | **Normotensive controls (n=19)** | **Adults with controlled arterial hypertension (n=38)** |
| Age, years | 56 (53, 59) | 59 (54, 64) |
| Sex, % female | 58 | 37 |
| Height, m | 1.71 (1.65, 1.80) | 1.76 (1.66, 1.82) |
| Weight, kg | 67.1 (62.2, 76.4) | 78.8 (64.8, 88.9) |
| Body fat, % | 14.9 (12.3, 20.1) | 19.5 (14.6, 24.9) |
| 24-h BP, sys/dia, mmHg | 119 (114, 123) / 81 (72, 83) | 130 (121, 134) / 84 (79, 89) |
| Stroke volume, mL | 73.4 (61.6, 85.4) | 69.75 (63.9, 77.5) |
| Mean 24-h HR, bpm | 61 (59, 71) | 71 (64, 77) |
| Mean nocturnal HR, bpm | 54 (47, 59) | 60 (52, 66) |
| Time since arterial hypertension diagnosis (years) | - | 6 (3.3, 11) |
| RER_max_ | 1.11 (1.08, 1.16) | 1.09 (1.05, 1.12) |
| APMHR, HR_max_ % of 210 - age | 110 (105, 116) | 112 (105, 115) |
| P_max_, Watts | 224 (172, 313) | 214 (162, 239) |
| Absolute V̇O_2peak_, L^.^min^-1^ | 2.53 (1.97, 3.44) | 2.57 (2.07, 2.97) |
| Relative V̇O_2peak_, mL^.^kg^-1.^min^-1^ | 36.0 (32.0, 46.0) | 33.0 (29.0, 37.0) |
| Data are presented as median (25^th^, 75^th^ percentile). Stroke volume is estimated from the 24-h blood pressure recording. Abbreviations: BP, blood pressure; APMHR, age-predicted maximum heart rate; P_max_, maximum power, RER_max_, maximum respiratory exchange ratio; sys, systolic, V̇O_2peak_, peak oxygen uptake. | | |

**24-hour blood pressure characteristics**

Supplementary table 2 presents characteristics of the 24-hour blood pressure measurements. Morning blood pressure surges were defined based on systolic blood pressure, calculated as the difference between the mean of the first two hours after wake-up and the average blood pressure of three readings centred on the lowest nighttime reading. Systolic and diastolic blood pressure dipping status were defined based on the percentage decline in mean nocturnal values relative to daytime values. Participants were classified as ‘yes’ if the difference exceeded 10%.^1^

| **Supplementary Table 2.** Characteristics of 24-hour blood pressure recording. | | | |
| --- | --- | --- | --- |
| **Variable** | **Normotensive controls (n=19)** | **Control group  (n=19)** | **Intervention group (n=19)** |
| Morning BP surges, mmHg | 16 (10, 25) | 18 (7, 22) | 10 (7, 19) |
| Systolic BP dipper, yes | 12 | 11 | 10 |
| Diastolic BP dipper, yes | 15 | 15 | 13 |
| Stroke volume, mL | 73 (62, 85) | 67 (63, 76) | 71 (64, 78) |
| Mean 24-h HR, bpm | 61 (59, 71) | 71 (62, 77) | 71 (66, 77) |
| Mean nocturnal HR, bpm | 54 (47, 59) | 60 (53, 67) | 59 (54, 66) |
| Data are presented as median (25^th^, 75^th^ percentile). Abbreviations: BP, blood pressure; HR, heart rate. | | | |

**Cross-sectional analyses**

| **Supplementary Table 3.** Effect for normotensive controls compared to adults with controlled arterial hypertension | |
| --- | --- |
| **Variables** | **Std. estimate [95% CI]** |
| V̇O₂ peak L/min | 0.44 [-0.10, 0.98] |
| V̇O₂ peak mL/kg/min | 0.4 [-0.21, 1.02] |
| V̇O₂ mL/kg/min at VT2 | 0.15 [-0.43, 0.72] |
| V̇O₂ mL/kg/min at VT1 | -0.67 [-1.28, -0.05] |
| OUES all data | 0.71 [0.17, 1.24] |
| OUES VT2 | 0.76 [0.22, 1.29] |
| OUES VT1 | 0.63 [0, 1.25] |
| V̇E/V̇CO₂ slope all data | 0.6 [0.03, 1.17] |
| V̇E/V̇CO₂ slope VT2 | -0.66 [-1.22, -0.1] |
| V̇E/V̇CO₂ slope VT1 | 0.47 [-0.13, 1.06] |
| PETCO₂ at VT2 | 0.39 [0, 0.77] |
| PETCO₂ at VT1 | 0.36 [0.02, 0.69] |
| V̇O₂/WR all data | 0.53 [-0.01, 1.14] |
| V̇O₂/WR VT2 | -0.34 [-0.82, 0.06] |
| V̇O₂/WR VT1 | 0.54 [-0.03, 1.15] |
| O₂ pulse at peak | 0.37 [-0.01, 0.73] |
| O₂ pulse at VT2 | 0.71 [0.20, 1.24] |
| O₂ pulse at VT1 | 0.19 [-0.19, 0.54] |
| Estimates are on the Z-scale and may be interpreted as interpreted as small (≥0.1 to <0.3), medium (≥0.3 to <0.5) and large (≥0.5).^2^ Positive Z-scores indicate higher values in normotensive adults than in patients with hypertension and vice versa. Abbreviations: O_2_ pulse, oxygen pulse; OUES, oxygen uptake efficiency slope; PETCO_2_, partial pressure of end-tidal carbon dioxide; V̇E/V̇CO_2_, ventilatory equivalent for carbon dioxide; V̇O_2_/WR, oxygen uptake to work rate ratio; V̇O_2peak_, peak oxygen uptake; VT1, ventilatory threshold 1; VT2, ventilatory threshold 2. | |

**Results of Partial Least Squares Regression (PLSR)**

In the PLSR analysis, O_2_ pulse and the V̇O_2_/WR slope were identified as the primary contributors to improvements in oxygen uptake efficiency slope (OUES). The loadings for the first component of the PLSR model showed that changes in O_2_ pulse at ventilatory threshold 2 (VT2) (loading: 0.850) and changes in V̇O_2_/WR slope (loading: 0.559) were the strongest contributors, while changes in PETCO_2_ at VT2 (loading: -0.238) and the V̇E/V̇CO_2_ slope (loading: -0.051) had weaker contributions. The model explained 73.1% of the variance with three components, based on cross-validated root mean square error of prediction results.

**Sensitivity analyses with additional adjustment for medication types**

Figure 2 show the results of the cross-sectional sensitivity analyses comparing normotensive adults to those with controlled arterial hypertension. These analyses were additionally adjusted for RAS inhibitors (ACE inhibitors or ARBs) and calcium channel blockers, included as two separate binary variables.

**Figure 2.** Estimates are on the Z-scale and may be interpreted as small (≥0.1 to <0.3), medium (≥0.3 to <0.5) and large (≥0.5).^2^ Positive Z-scores indicate higher values in normotensive adults than in adults with hypertension and vice versa. Abbreviations: O_2_ pulse, oxygen pulse; OUES, oxygen uptake efficiency slope; PETCO_2_, partial pressure of end-tidal carbon dioxide; V̇E/V̇CO_2_, ventilatory equivalent for carbon dioxide; V̇O_2_/WR, oxygen uptake to work rate ratio; V̇O_2peak_, peak oxygen uptake; VT1, ventilatory threshold 1; VT2, ventilatory threshold 2.

**References**

1.	Kario K, Pickering TG, Umeda Y, Hoshide S, Hoshide Y, Morinari M, Murata M, Kuroda T, Schwartz JE, Shimada K. Morning Surge in Blood Pressure as a Predictor of Silent and Clinical Cerebrovascular Disease in Elderly Hypertensives. *Circulation*. 2003;107(10):1401–1406. doi:doi:10.1161/01.CIR.0000056521.67546.AA

2.	Cohen J. *Statistical power analysis for the behavioral sciences*. Academic press; 2013.
